# Development and validation of machine learning models for predicting pancreatic injury observed after tigecycline initiation: a retrospective real-world study

**DOI:** 10.3389/fphar.2026.1930240

**Published:** 2026-09-29

**Authors:** Fulong Huang, Jiayu Chen, Xiangdong Wang, Chunping Gu, Liren Li, Ping Zheng

**Affiliations:** 1 Department of Pharmacy, Nanfang Hospital, Southern Medical University, Guangzhou, China; 2 Clinical Pharmacy Center, Nanfang Hospital, Southern Medical University, Guangzhou, China; 3 Department of Pharmacy, Nanhai Guicheng Hospital, Foshan, China

**Keywords:** lightgbm, machine learning, pancreatic injury, pharmacoepidemiology, tigecycline

## Abstract

**Background:**

Tigecycline is widely used for severe infections and infections caused by multidrug-resistant pathogens. Although pancreatic injury observed after tigecycline initiation is relatively uncommon, it may adversely affect patient prognosis. Currently, clinical management mainly relies on post-treatment monitoring, and reliable tools for early risk prediction are lacking. This study aimed to develop and internally validate an interpretable machine learning model for predicting pancreatic injury observed after tigecycline initiation using real-world clinical data.

**Methods:**

This single-center retrospective study included patients who received tigecycline for at least 3 consecutive days at Nanfang Hospital, Southern Medical University, between January 2020 and March 2024. The primary outcome was pancreatic injury observed after tigecycline initiation, defined as serum amylase >405U/L and/or serum lipase >180 U/L within 7 days after tigecycline initiation. Demographic characteristics, comorbidities, concomitant medications, tigecycline treatment information, and baseline laboratory parameters were collected. Missing values were imputed using K-nearest neighbors imputation, and class imbalance was addressed using SMOTE. The dataset was randomly divided into training and test sets at an 8:2 ratio. Eight machine learning algorithms were developed and compared. Model performance was evaluated using AUC, accuracy, sensitivity, specificity, positive predictive value, negative predictive value and F1 score. SHAP analysis was used for model interpretation.

**Results:**

A total of 623 patients were included, of whom 146 (23.4%) developed pancreatic injury. Among the evaluated algorithms, LightGBM had the highest point-estimate AUC. The final 7-feature LightGBM model achieved an AUC of 0.726, accuracy of 0.728, sensitivity of 0.379, specificity of 0.833, PPV of 0.407, NPV of 0.816, and F1 score of 0.393 on the held-out test set. These results indicate moderate discrimination but limited sensitivity.

**Conclusion:**

With relatively high specificity and negative predictive value, this model may provide preliminary risk stratification information and assist targeted pancreatic enzyme monitoring during tigecycline therapy, particularly for identifying patients at lower predicted risk. However, prospective external validation is required before routine clinical implementation.

## Introduction

Antimicrobial resistance (AMR) is recognized as one of the most pressing threats to modern medicine and global public health systems (Marino et al., 2025). It poses a severe and escalating challenge worldwide, contributing to increased risks of morbidity and mortality, and is identified by the World Health Organization (WHO) as a critical public health concern (Kavya et al., 2024). Against this backdrop, tigecycline is a glycylcycline antibiotic that was approved by the U.S. Food and Drug Administration in June 2005 for the treatment of complicated skin and skin structure infections, complicated intra-abdominal infections, and community-acquired pneumonia (Liu et al., 2021; Zhou et al., 2022). It is not affected by most bacterial resistance mechanisms, including tetracycline resistance (Nørskov-Lauritsen et al., 2009). Consequently, tigecycline is considered a last-resort antibiotic against severe infections.

Tigecycline is well tolerated. In clinical trials and post-marketing experience, the safety summary of the use of tigecycline shows that nausea and vomiting are the most common adverse reactions (Guo et al., 2022). However, case reports have demonstrated that tigecycline may induce pancreatic injury and even pancreatitis, characterized by severe abdominal pain accompanied by elevated serum amylase (AMS) and lipase (LPS) levels (Hung et al., 2009; Hemphill and Jones, 2016; Lin et al., 2018). Pancreatitis is a severe inflammatory condition that is associated with abdominal pain, nausea, vomiting, and potentially multiple organ failure (Tenner et al., 2024; Pázmány et al., 2025). It often results in prolonged hospitalization, increased healthcare costs, and adversely affects the overall prognosis of patients. Currently, there are no reliable predictive tools available to identify which patients are at risk of pancreatic injury following tigecycline administration. Clinical management predominantly relies on post-administration monitoring rather than pre-treatment prediction, representing a reactive, “post-event” management strategy that cannot facilitate early warning or intervention.

Previous studies on adverse drug reactions to tigecycline have primarily relied on case reports, small-sample retrospective analyses, or post-marketing surveillance, with the main objective of confirming the existence and incidence of these events (Okon et al., 2013; Turan Gökçe et al., 2024; Feng et al., 2025). These conventional research approaches are inadequate for integrating complex patient characteristics—such as comorbidities, concomitant medications, and dynamic changes in laboratory parameters—to accurately predict individual patient risk. Therefore, this study aimed to develop and validate an interpretable machine learning-based prediction model for the early identification of patients at risk of pancreatic injury observed after tigecycline initiation. By using routinely available clinical variables, this model may support individualized risk stratification and proactive monitoring during tigecycline therapy.

## Methods

### Patients

This single-center retrospective study was conducted at Nanfang Hospital, Southern Medical University. Patients who received tigecycline therapy between January 2020 and March 2024 were included. The inclusion criteria were as follows: (1) treatment with tigecycline for at least 3 consecutive days; and (2) availability of serum amylase and/or lipase measurements before tigecycline initiation and during follow-up after tigecycline administration. Patients were excluded if they met any of the following criteria: (1) serum amylase or lipase levels exceeding three times the upper limit of normal before tigecycline initiation; (2) more than 30% missing values among candidate predictors; (3) age younger than 18 years; or (4) a documented history or diagnosis of acute pancreatitis, chronic pancreatitis, pancreatic cancer, or other pancreatic diseases before tigecycline treatment. The patient screening process is shown in Figure 1. After applying these criteria, a total of 623 eligible patients were included in the final analysis.

**FIGURE 1:**
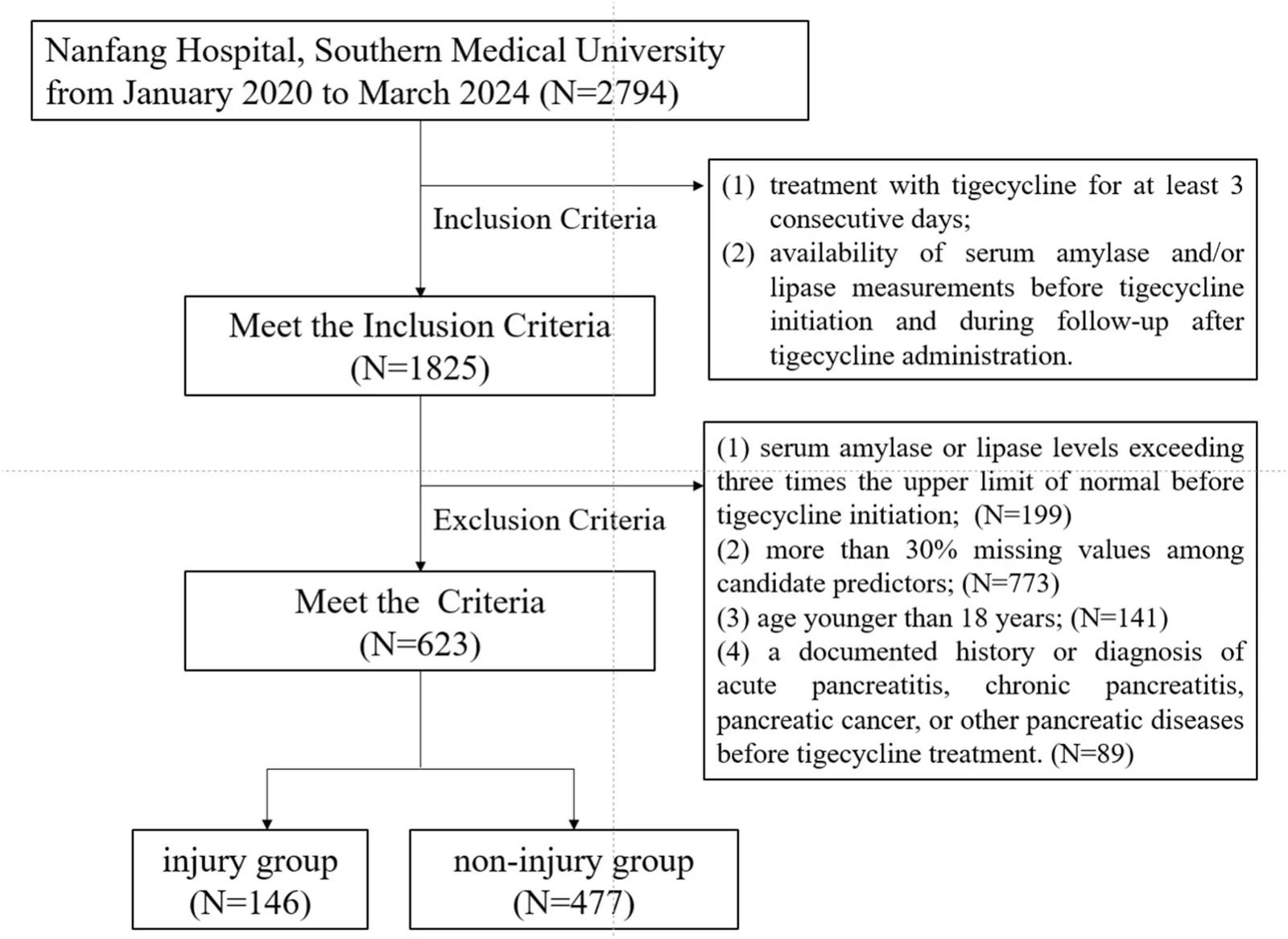
Flowchart of included patients.

### Data collection and definition

Using an electronic medical record system, we collected data on 623 eligible patients. Predictors were extracted from data available before the initiation of tigecycline therapy to minimize information leakage and to reflect real-world pre-treatment risk assessment. The dataset included four main categories of variables: (1) demographic information (such as age, BMI, sex, smoking and drinking history); (2) medication use (such as diuretic, olanzapine, metformin, and other drugs); (3) comorbidities (such as fatty liver, cholecystitis, and gallstones); and (4) laboratory parameters (such as renal function, liver function, and routine blood tests). Serum amylase and lipase levels were monitored during hospitalization. The primary outcome was pancreatic injury observed after tigecycline initiation, defined as serum amylase >405 U/L and/or serum lipase >180 U/L within 7 days after tigecycline initiation. This endpoint included both asymptomatic enzyme elevation and clinically diagnosed acute pancreatitis. Patients meeting either criterion were classified into the pancreatic injury group, whereas those whose serum amylase and lipase both remained below these thresholds during the 7-day follow-up were classified into the non-injury group. The 7-day window was selected to focus on early-onset pancreatic injury and was informed by previous reports showing a median onset of approximately 7 days for tigecycline-associated pancreatitis; in addition, restricting the observation period to the early treatment phase improved comparability of biochemical monitoring across hospitalized patients. However, because tigecycline-associated pancreatitis may also occur beyond 7 days, this definition may not capture delayed-onset events. In addition, given that serum pancreatic enzyme elevation can be influenced by baseline pancreatic status and renal clearance capacity, baseline amylase and lipase levels were collected as candidate predictors, and patients with marked baseline pancreatic enzyme elevation (>3 times the upper limit of normal) or previous pancreatic diseases were excluded. Accordingly, the model should be interpreted as predicting pancreatic injury observed after tigecycline initiation rather than establishing a causal diagnosis of tigecycline-induced pancreatitis.

### Data processing

#### Dataset splitting

The retrospectively collected cohort data were randomly divided into a training set (80%) and a test set (20%). The training set was used for model development and fitting of all preprocessing procedures, while the test set was reserved exclusively for internal validation. To eliminate data leakage, all preprocessing steps (including missing value imputation, feature scaling, and class balancing) were fitted solely on the training set; the test set was transformed only using parameters learned from the training set and did not participate in any fitting process.

#### Missing value imputation

Missing data in medical datasets can introduce bias or inaccuracies in subsequent data analysis and machine learning models. To address this, we compared the model performance of multiple missing value imputation methods, including K nearest neighbors (KNN) imputation, median imputation, and multiple imputation, based on cross-validation within the training set (Supplementary Table S2). Ultimately, the KNN imputation method exhibited the best performance. Therefore, the KNN imputer was fitted exclusively on the training set, and then applied to impute missing values in both the training and test sets. The optimal imputation result was achieved when K = 3, as determined by grid search with cross-validation within the training set.

#### Data standardization

A standard scaler was applied to normalize the imputed data by transforming it into a standard normal distribution with a mean of 0 and a standard deviation of 1. This process ensures uniformity in the scales across different features, thereby mitigating bias introduced by discrepancies in feature magnitudes and enhancing both data stability and model training accuracy.

#### Unbalanced data processing

To address the challenge of data imbalance between the injury and non-injury groups in the training set, the K-nearest neighbors-based Synthetic Minority Over-sampling Technique (SMOTE) was applied. This method synthesizes minority class samples by interpolating between adjacent minority instances until the desired sample size for the minority class is achieved. SMOTE was implemented exclusively on the scaled training set, and the test set retained its original class distribution without any over-sampling manipulation. This approach enhanced the model’s generalization capability and classification accuracy, while reducing the risk of overfitting and ensuring unbiased performance estimation during validation.

### Model development and comparison

Eight machine learning algorithms—Gradient Boosting Classifier (GBC), Light Gradient Boosting Machine (LightGBM), Multilayer Perceptron (MLP), Support Vector Machine (SVM), Voting Classifier, Logistic Regression, Extreme Gradient Boosting (XGBoost), and Random Forest (RF)—were employed to construct predictive models based on the training data, followed by internal validation on the test data. To prevent model overfitting, both five-fold and ten-fold cross-validation were performed on the training set. The test set was used exclusively for final internal validation. Finally, the performance of the models was compared to identify the optimal model.

In this study, LightGBM are specifically selected due to its optimal discriminatory performance. LightGBM is a gradient boosting framework that utilizes histogram-based algorithms and a leaf-wise tree growth strategy. Designed for large-scale data, it offers fast training speed, low memory consumption, and high accuracy, with support for GPU acceleration and distributed computing, making it ideal for handling high-dimensional sparse features.

### Optimal feature selection

Feature importance was ranked using the Random Forest (RF) algorithm. To optimize feature selection, the number of features was iteratively reduced from 40 to 7, in decrements of 3 features per step, based on their importance ranking. Throughout this process, the predictive performance of the resulting feature subsets was evaluated.

### Model evaluation indexes

To evaluate the predictive performance of the models, multiple metrics were employed, including the area under the receiver operating characteristic curve (AUC), accuracy, sensitivity, specificity, positive predictive value (PPV), negative predictive value (NPV) and F1 score. For binary classification, the default probability threshold of 0.50 was applied, with predicted probabilities ≥0.50 classified as pancreatic injury and probabilities <0.50 classified as non-injury. The formulas for each evaluation metric are provided below.

$$Accuracy=\frac{TP+TN}{TP+TN+FP+FN}$$

$$Sensitivity=recall=\frac{TP}{TP+FN}$$

$$Specificity=\frac{TN}{TN+FP}$$

$$PPV=precision=\frac{TP}{TP+FP}$$

$$NPV=\frac{TN}{TN+FN}$$

$$F1\;score=2\times \frac{TP}{TP+\frac{1}{2}\left(FP+FN\right)}$$

TP: True Positive; TN: True Negative; FP: False Positive; FN: False Negative.

### Model explanation

SHAP is an interpretability analysis method based on cooperative game theory, designed to explain machine learning models. Its core principle involves decomposing the model’s predictive output into contribution values attributable to each feature. In this study, for each input sample, all possible combinations of features were considered to form a comprehensive feature space. The marginal contribution of each feature was then calculated across different combinations, representing the change in the model’s output when that feature was added. The Shapley value for each feature was computed using a weighted average of its marginal contributions, with the weights being the number of possible feature combinations and their corresponding probabilities. This value indicates the direction and magnitude of a feature’s contribution to the model output for a given prediction, but it does not establish causality or identify true protective or risk factors.

### Statistical analysis

All statistical analyses were performed using IBM SPSS (version 27.0) and Python (version 3.10.0). Categorical data are presented as frequency and percentage [n (%)], with comparisons between groups conducted using the chi-square test or Fisher’s exact test, as appropriate. Continuous variables are summarized as the median (interquartile range, IQR). The Mann–Whitney U test was employed to compare continuous variables between groups. To further evaluate the association between tigecycline maintenance dose and pancreatic injury, multivariable logistic regression analysis was performed with adjustment for clinically relevant potential confounding factors, including age, sex, PCT, eGFR, baseline amylase, baseline lipase, and glucocorticoid use. Associations are reported as odds ratios (ORs) with 95% confidence intervals (CIs). A P-value of <0.05 was considered statistically significant.

## Results

### Patient baseline characteristics

A total of 623 patients were enrolled, among whom 146 (23.4%) were assigned to the injury group and 477 (76.6%) to the non-injury group. Among the 146 patients with pancreatic injury, 21 had isolated amylase elevation, 87 had isolated lipase elevation, and 38 had both enzymes elevated. Thirty-three patients met the revised Atlanta criteria for acute pancreatitis, accounting for 5.3% of all patients; among these 33 patients, 6 had isolated amylase elevation, 11 had isolated lipase elevation, and 16 had both enzymes elevated. These findings indicate that pancreatic injury observed after tigecycline initiation encompasses a heterogeneous spectrum, ranging from isolated biochemical enzyme abnormalities to clinically defined acute pancreatitis. No statistically significant differences were observed between the two groups in demographic factors, including sex (male proportion approximately 70% in both groups, P = 0.975), median age (56.0 years vs. 59.0 years, P = 0.542), and lifestyle habits (smoking and alcohol consumption, P > 0.05). Regarding comorbidities, no significant differences were detected (all P > 0.05). In terms of concomitant medications, the injury group exhibited a significantly higher rate of glucocorticoid use (80.82% vs. 68.34%, P = 0.005). For laboratory parameters, the injury group demonstrated a significantly lower estimated glomerular filtration rate (eGFR) (56.92 vs. 89.66 mL/min/1.73 m^2^, P < 0.001) and higher levels of inflammatory markers, particularly procalcitonin (PCT) (1.32 vs. 0.59 ng/mL, P < 0.001). After Bonferroni correction for multiple testing, the differences in eGFR and PCT between the injury and non-injury groups remained statistically significant (both adjusted P < 0.001). Regarding tigecycline treatment parameters, the maintenance dose was significantly higher in the injury group (P = 0.0032), while no significant differences were found in loading dose. When stratified by maintenance dose category, the incidence of pancreatic injury was 40.0% (2/5), 17.9% (52/291), and 28.1% (92/327) among patients receiving 25 mg, 50 mg, and 100 mg, respectively. Compared with the 50 mg maintenance dose, patients receiving 100 mg had a higher risk of pancreatic injury (OR: 1.80, 95% CI: 1.22-2.64, P = 0.003). These findings suggest an association between higher maintenance dose and pancreatic injury. However, this association should be interpreted cautiously because dose selection may be influenced by underlying disease severity. To account for potential confounding, we further examined the association using multivariable logistic regression. After adjustment for age, sex, PCT, eGFR, baseline amylase, baseline lipase, and glucocorticoid use, a maintenance dose of 100 mg remained statistically associated with pancreatic injury compared with 50 mg (OR = 1.51, 95% CI: 1.21–1.82, P = 0.01). However, because direct measures of illness severity and infection complexity were unavailable, this association should not be interpreted as independent evidence of a dose-related causal effect. Further details are provided in Supplementary Table S3.

### Sensitivity analysis

KNN sensitivity experiment: In KNN, the choice of the K value is critical to the final prediction performance, so we conducted a hyperparameter sensitivity analysis (Supplementary Table S4; Supplementary Figure S1A). The results clearly indicated that the model achieves the best overall performance when K = 3.

Oversampling sensitivity experiment: To evaluate the sensitivity of the model to imbalanced training data inputs, this study employed the SMOTE oversampling method to examine different values of the k_neighbors parameter (Supplementary Table S5; Supplementary Figure S1B). The results showed that k_neighbors = 3 yielded superior performance and achieved satisfactory outcomes.

### Model development

Eight machine learning algorithms were trained using all 40 routine test features, and their predictive performance was compared, as presented in Table 1. Among the eight algorithms, LightGBM achieved the highest AUC (0.730), demonstrating superior overall performance. The logistic regression model yielded the lowest AUC. (Figure 2).

**TABLE 1:** Performance of the 8 ML models.

Model	AUC	AC	SE	SP	NPV	PPV	F1
LightGBM	0.730	0.768	0.310	0.906	0.813	0.500	0.383
GBC	0.647	0.712	0.207	0.865	0.783	0.316	0.250
Random forest	0.630	0.776	0.241	0.938	0.804	0.538	0.333
Voting classifier	0.712	0.776	0.276	0.927	0.809	0.533	0.364
XGBoost	0.689	0.776	0.310	0.917	0.815	0.529	0.391
MLP	0.630	0.712	0.276	0.843	0.794	0.348	0.308
SVM	0.588	0.664	0.310	0.771	0.787	0.290	0.300
LR	0.549	0.584	0.414	0.635	0.782	0.255	0.316

ML: machine learning; AUC: area under the receiver operating characteristic curve; SE: sensitivity; SP: specificity; PPV: positive predictive value; NPV: negative predictive value; AC: accuracy; F1: F1 score; GBC: gradient boosting classifier; LightGBM: light gradient boosting machine; MLP: Multi-Layer Perceptron; SVM: support vector machine; XGBoost: eXtreme gradient boosting; LR: logistic regression.

**FIGURE 2:**
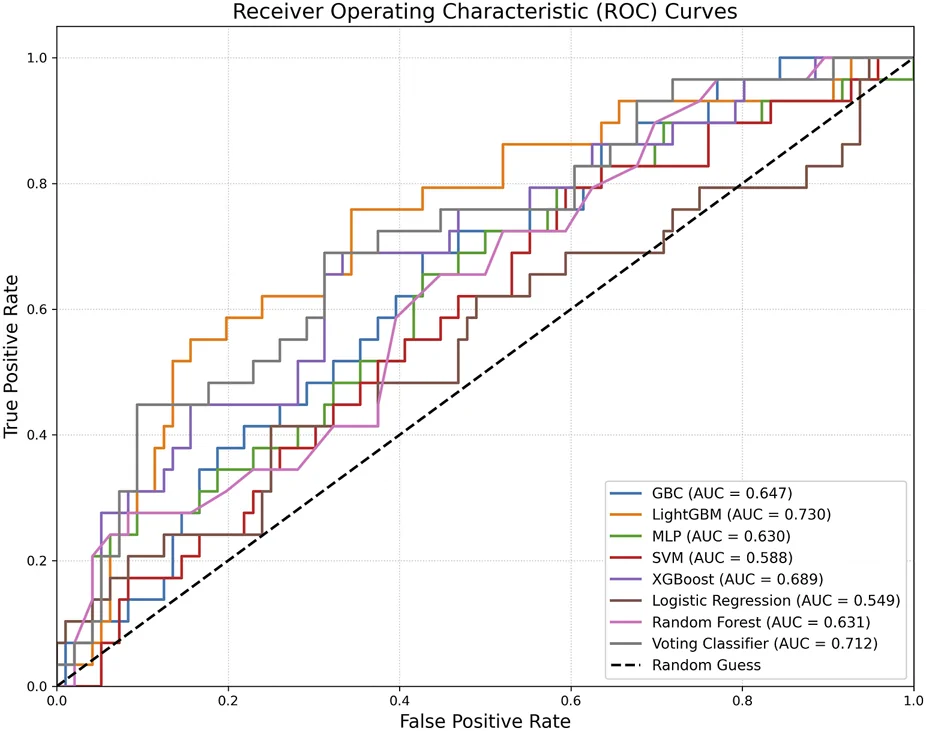
ROC curves of the diagnostic models generated by 8 ML algorithms. ML: machine learning; ROC: receiver operating characteristic; AUC: the area under the receiver operating characteristic curve; GBC: gradient boosting classifier; LightGBM: light gradient boosting machine; MLP: Multi-Layer Perceptron; SVM: support vector machine; XGBoost: eXtreme gradient boosting; RF: random forest.

### Feature optimization

The LightGBM model was subsequently optimized (Figure 3A; Supplementary Table S6). As illustrated in Figure 3B, the full-feature model (40 features) achieved the highest area under the curve (AUC) of 0.730 (SD ± 0.05). Based on the 1-SE rule, the most parsimonious model within one standard error of this maximum was selected, which contained 7 features and yielded an AUC of 0.726, demonstrating comparable discrimination to the full-feature model while using substantially fewer features. These 7 features included one medication-related variable and 6 laboratory parameters (Supplementary Figure S2). Accordingly, the LightGBM model incorporating these 7 features was selected as the final model.

**FIGURE 3:**
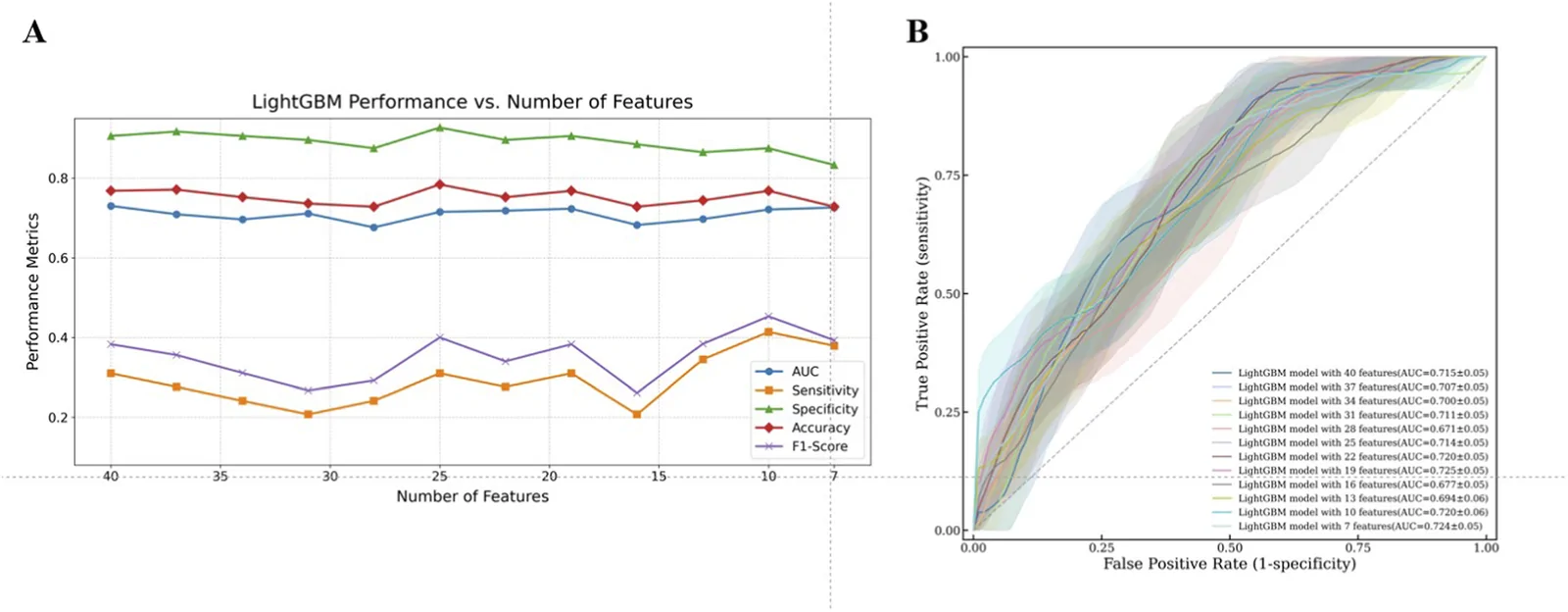
Model evaluation of LightGBM based on the ROC curve. **(A)** AUC, specificity, accuracy, and NPV of LightGBM model with different feature combinations. **(B)** ROC curves of LightGBM model with different feature combinations. ROC: receiver operating characteristic; AUC: area under the receiver operating characteristic curve; LightGBM: light gradient boosting machine.

To prevent overfitting, five-fold and ten-fold cross-validation were performed on the LightGBM model, with the results visualized in Supplementary Figure S3.

### Clinical utility of the final ML model

As shown in Figure 4, the DCA demonstrates that the net benefit of the final 7-feature LightGBM model is consistently higher than both the treat-all and treat-none strategies across a threshold probability range of 0.12–0.40. The model’s performance over this clinically relevant range suggests favorable clinical utility, suggesting potential utility for individualized monitoring strategies. However, because model training incorporated SMOTE and probability calibration was not formally performed, these decision curves should be interpreted as exploratory rather than definitive evidence of clinical benefit.

**FIGURE 4:**
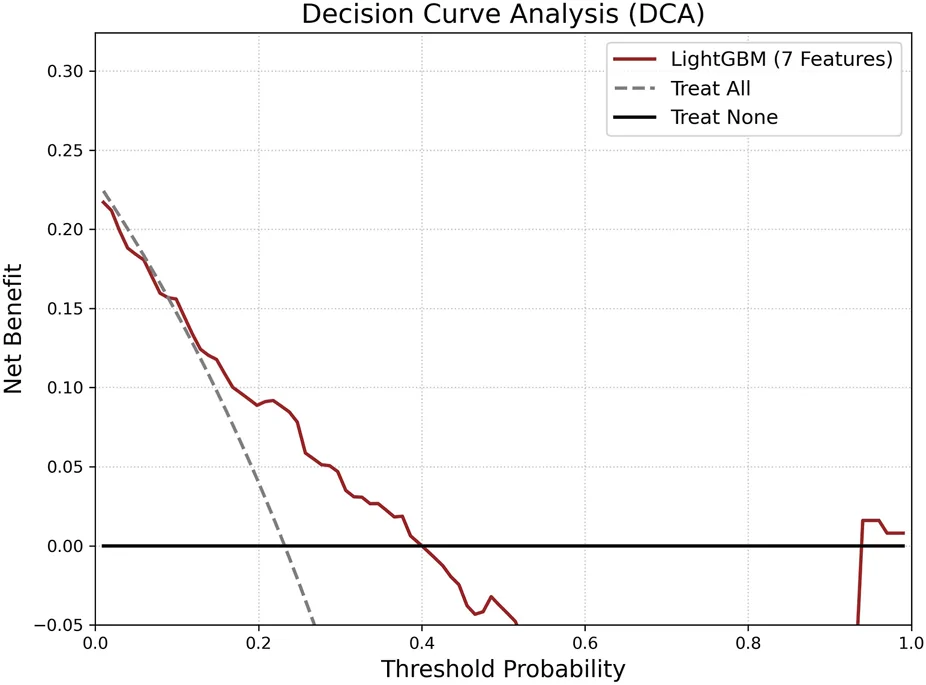
DCA of the final LightGBM model. DCA: decision curve analysis; LightGBM: light gradient boosting machine.

### Model interpretation

The interpretability of the optimal LightGBM model was examined using SHAP (SHapley Additive exPlanations) to elucidate feature contributions and their directional influences on predictions. Figure 5A presents the feature importance ranking based on mean absolute SHAP values. Maintenance dose was the most important predictor, followed by Procalcitonin (PCT) and eGFR. Figure 5B provides a SHAP summary plot detailing the effects of the 7 features across individual samples. Each point corresponds to a sample’s feature value, plotted horizontally according to its SHAP value (impact direction and magnitude) and colored by the original feature magnitude (blue for low, red for high). The results indicated that higher maintenance dose and PCT count levels were associated with an increased risk of pancreatic injury, whereas a higher eGFR exerted the opposite effect. This plot visually demonstrates how different feature values differentially drive the model’s risk predictions. Additionally, SHAP dependence plots for each feature are shown in Supplementary Figure S4.

**FIGURE 5:**
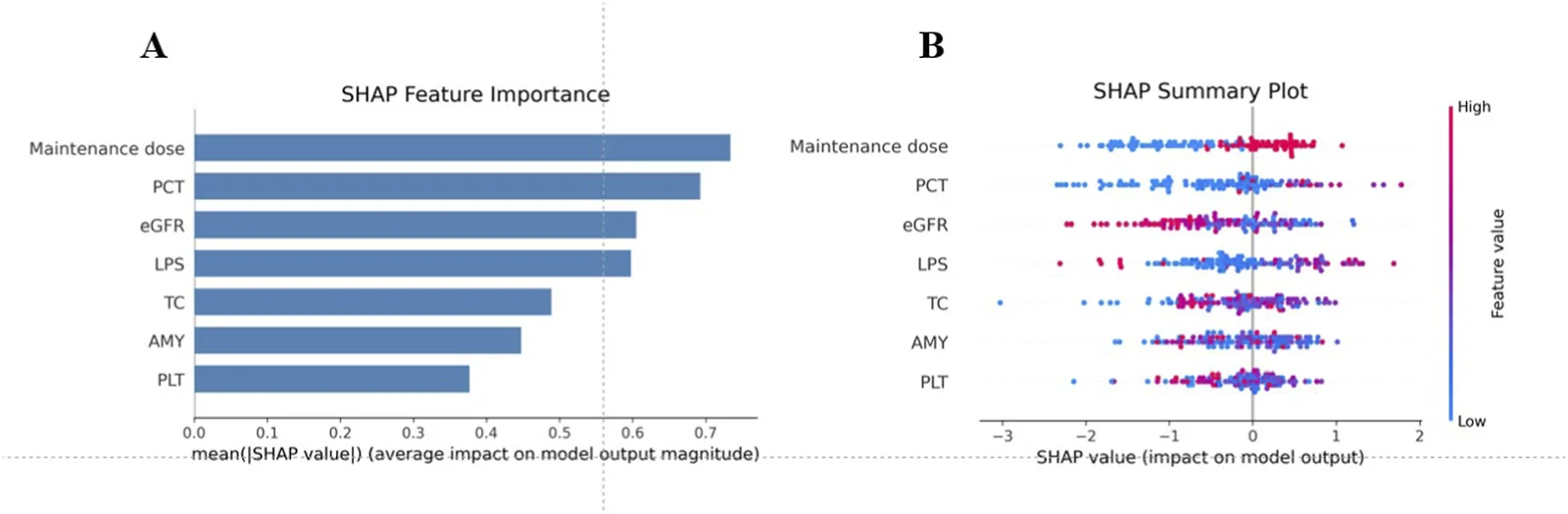
Global explanation of the model by SHAP method. **(A)** Feature importance matrix plot. **(B)** SHAP summary plot. PCT: procalcitonin; eGFR: estimated glomerular filtration rate; LPS: lipase; TC: total cholesterol; AMY: amylase; PLT: platelets.

The SHAP local explanations further elucidate how each feature influences the probability of being diagnosed with pancreatic injury for each patient. Figure 6A and B highlight the SHAP values of key variables for a representative patient in the injury group and another representative patient in the non-injury group, respectively. The SHAP values for all patients in the training set are presented in Figure 6C.

**FIGURE 6:**
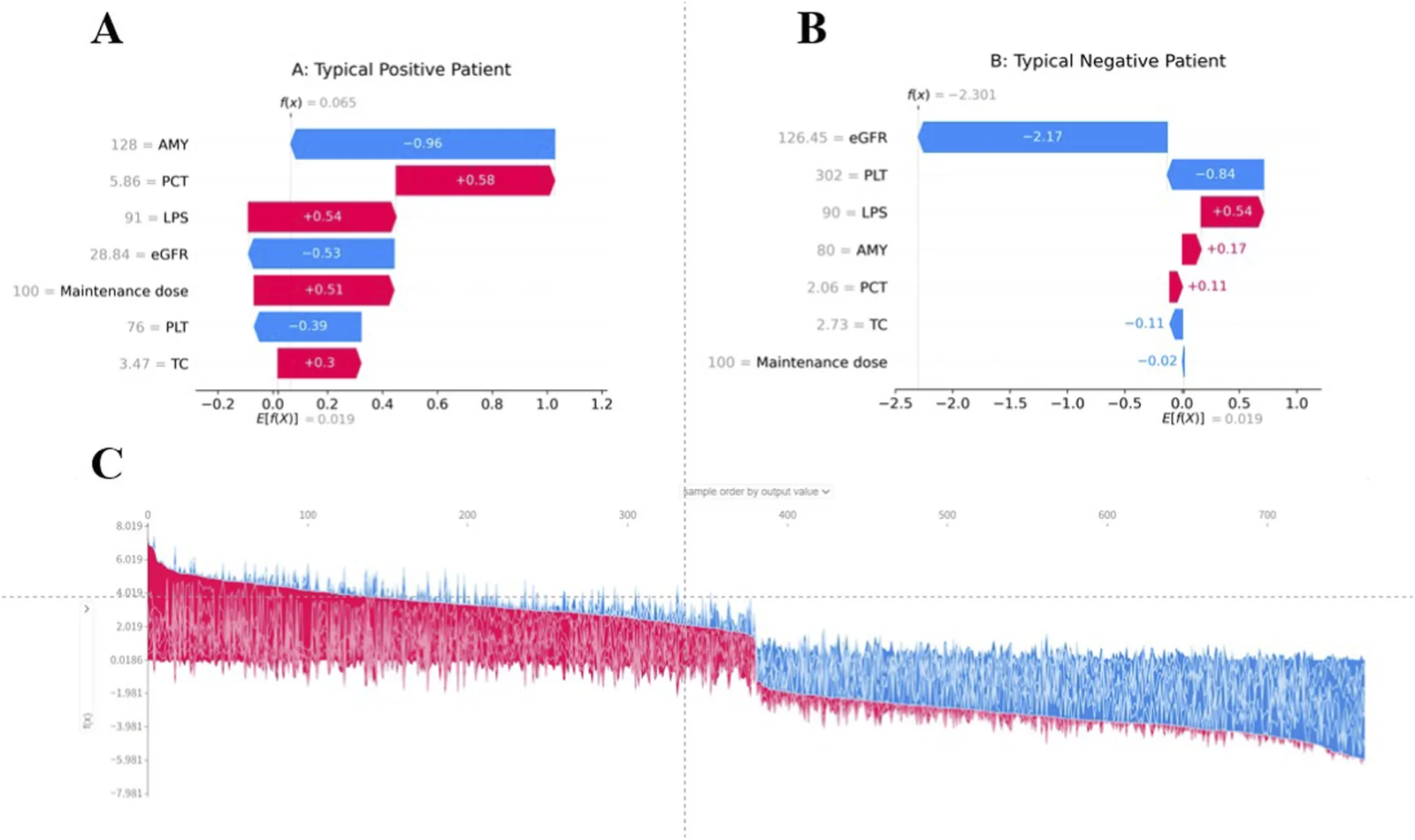
Local explanation of the model by SHAP method. **(A,B)** SHAP values of two typical patients from the injury group **(A)** and the non-injury group **(B)** are illustrated with their most important variables. **(C)** SHAP values for all patients in the training set. SHAP: SHapley Additive explanation; PCT: procalcitonin; eGFR: estimated glomerular filtration rate; LPS: lipase; TC: total cholesterol; AMY: amylase; PLT: platelets.

## Discussion

In this study, predictive models for pancreatic injury observed after tigecycline initiation were developed using eight machine learning algorithms and clinical laboratory variables. Upon comparison, the LightGBM algorithm exhibited the optimal performance, with an AUC of 0.730 and high accuracy/specificity. After feature selection, the final 7-feature LightGBM model achieved an AUC of 0.726, close to that of the full-feature model, with relatively high specificity (83.3%) but limited sensitivity. Although feature selection improved model parsimony without substantially reducing discrimination, the low sensitivity indicates that a substantial proportion of patients who subsequently developed pancreatic injury would not be identified. Therefore, the current model should be considered an auxiliary risk-stratification approach rather than a standalone decision-making tool. SHAP analysis was primarily used to characterize the contribution of individual predictors rather than to establish causal risk factors.

The findings of the present study are highly consistent with clinical observations reported in the existing literature, while representing a marked advancement in both depth of investigation and methodological approach. To date, tigecycline-induced elevations in pancreatic enzymes have been primarily documented in case reports as rare adverse reactions (Lin et al., 2018; Wang et al., 2021; Yazirli et al., 2021), highlighting the need for clinical vigilance regarding its potential pancreatic toxicity during therapeutic use, with causality typically inferred from symptomatic improvement upon drug discontinuation (McGovern et al., 2014; Hemphill and Jones, 2016; Tan et al., 2023). However, a systematic risk assessment model has not been established in the literature, and most reports are based on retrospective observations lacking quantitative analysis of risk factors. Notably, meta-analyses of large-scale clinical trial data have reported an incidence of tigecycline-associated pancreatitis of less than 1% (McGovern et al., 2014; Tan et al., 2023), although an increased risk has been observed in the context of concomitant medication use or among specific patient populations (e.g., kidney transplant recipients, patients with cystic fibrosis) (Badalov et al., 2007; Hemphill and Jones, 2016; Pan et al., 2023). In contrast, the present study employed machine learning techniques for the first time to integrate multidimensional data, including patient demographics and laboratory parameters. Missing values were addressed using KNN imputation, dataset imbalance was corrected *via* the SMOTE, and eight predictive models, including LightGBM, were developed. Through feature importance analysis and SHAP interpretation, key high-risk features were identified, enabling early warning of tigecycline-induced pancreatic injury. This approach provides clinicians with a quantitative decision-support tool, moving beyond reliance on subjective judgment or monitoring of isolated biochemical parameters.

Among the eight algorithms compared, the LightGBM model exhibited the best overall performance over the other machine learning approaches, primarily attributable to its unique algorithmic architecture and efficient adaptability to feature processing. First, the histogram-based decision tree construction strategy employed by LightGBM discretizes continuous features into histogram bins, substantially reducing the number of data scans and thereby markedly improving computational efficiency and memory utilization (Yan et al., 2021; Ke et al., 2017). Second, LightGBM adopts a leaf-wise growth strategy, in contrast to the traditional level-wise approach, which enables a faster reduction in the loss function value and helps prevent overfitting while maintaining model accuracy. Compared with models sensitive to feature scale and distribution, such as support vector machine (SVM) and multilayer perceptron (MLP), LightGBM demonstrates greater robustness to standardized features (Pinheiro et al., 2025). In summary, these algorithmic optimizations elucidate why the LightGBM model achieved the highest AUC value in predicting the risk of pancreatic injury observed after tigecycline initiation and outperformed conventional machine learning models across multiple dimensions.

SHAP analysis identified maintenance dose, PCT, and eGFR as the most influential features in the final model. Higher maintenance dose and PCT values were associated with higher predicted risk, whereas higher eGFR was associated with lower predicted risk. Baseline pancreatic enzyme levels, particularly lipase, also contributed to model prediction. These findings should be interpreted as predictive associations rather than causal relationships, as SHAP values quantify the contribution of individual features to model predictions but do not establish causal effects (Ponce-Bobadilla et al., 2024). The contribution of PCT may partly reflect the severity of systemic infection and inflammation, as elevated PCT concentrations have been associated with greater disease severity and poorer outcomes in patients with sepsis (Wacker et al., 2013; Liu et al., 2015). The inverse association between eGFR and predicted risk is also consistent with previous observations that renal insufficiency may be relevant to pancreatic injury observed after tigecycline initiation. Renal insufficiency has been identified as an independent risk factor for tigecycline-induced pancreatitis (Tan et al., 2023), and severe renal impairment has been shown to reduce tigecycline clearance and increase systemic exposure (Su et al., 2024). In addition, impaired renal function may itself contribute to elevated pancreatic enzyme concentrations through reduced enzyme clearance (Feher et al., 2024). The contribution of maintenance dose may therefore reflect not only drug exposure but also treatment intensity, indication, and underlying clinical severity; importantly, a direct dose–toxicity relationship for pancreatic injury observed after tigecycline initiation has not been established (Pan et al., 2023). Baseline amylase and lipase, TC, and PLT provided additional predictive information. The contribution of baseline pancreatic enzymes is clinically plausible; however, elevations in these biomarkers are not specific to pancreatic injury and may also occur in the setting of renal dysfunction, critical illness, or other systemic conditions. Previous reports of tigecycline-induced pancreatitis have consistently described increases in serum amylase and/or lipase, although most available evidence is derived from retrospective studies and case-based literature (Badalov et al., 2007; Pan et al., 2023). By contrast, the biological basis for the contributions of TC and PLT remains uncertain. Therefore, these SHAP findings are primarily useful for understanding how individual variables contribute to model predictions and should not be interpreted as evidence of specific pathophysiological mechanisms (Ponce-Bobadilla et al., 2024).

The current model holds significant value for risk assessment prior to tigecycline treatment. First, for patients with elevated PCT levels or reduced eGFR, the model can identify high-risk characteristics in advance, thereby assisting clinicians in implementing risk stratification before drug administration. The current model may provide preliminary risk stratification information before tigecycline administration. For patients with predicted higher risk characteristics, the model may help clinicians identify individuals who could benefit from closer pancreatic enzyme monitoring during therapy. However, whether model-guided monitoring strategies or dose modification approaches can improve clinical outcomes or reduce the risk of pancreatic injury requires prospective evaluation. Furthermore, SHAP-based interpretation provides insight into how individual variables contribute to model predictions and may improve the transparency of machine learning applications in clinical research (Pan et al., 2025). Nevertheless, these predictive contributions should not be interpreted as direct evidence for therapeutic decision-making. The current model should therefore be considered a supportive risk assessment tool rather than a tool for guiding individualized tigecycline dosing strategies. Future prospective studies are required to determine whether integrating such prediction models into clinical workflows can provide meaningful benefits for patient management.

Although the predictive model developed in this study has achieved preliminary success, several limitations should be acknowledged. First, the data were derived from a single center with a relatively limited sample size. Although SMOTE oversampling was employed to address class imbalance, the model may still be subject to selection bias, limiting its generalizability to different populations and healthcare settings. Second, the retrospective study design inherently precluded comprehensive control of all potential confounding factors. Although the association between maintenance dose and pancreatic injury was evaluated after adjustment for several clinically relevant variables, residual confounding by indication remains possible, as higher tigecycline doses may have been preferentially administered to patients with more severe infections or critical illness. Direct measures of illness severity, such as ICU admission, SOFA/APACHE II scores and mechanical ventilation, were not consistently available and therefore could not be included in the adjusted analysis. In addition, the classification of pancreatic injury was based primarily on biochemical enzyme elevation, which may be influenced by baseline pancreatic enzyme status and impaired renal clearance. Although baseline amylase, lipase, and eGFR were incorporated into the model and patients with pre-existing pancreatic diseases or marked baseline enzyme elevations were excluded, residual misclassification cannot be completely ruled out; future studies adopting stricter exclusion criteria are warranted to verify our conclusions. Other unmeasured variables, including dietary patterns and undocumented underlying biliary tract disorders, may also introduce residual confounding and affect model performance. Third, this study primarily focused on elevations in pancreatic enzyme levels and did not further differentiate between asymptomatic elevations and clinically defined acute pancreatitis, which may introduce bias in the clinical specificity of the model. Finally, formal calibration assessment was not performed because of the limited size of the internal validation cohort. Therefore, the reported discrimination estimates should be interpreted cautiously. In addition, the model lacks an independent external validation cohort, and its predictive performance in real-world settings warrants further investigation.

## Conclusion

In conclusion, an interpretable machine learning model was developed and internally validated for predicting pancreatic injury observed after tigecycline initiation. The model demonstrated moderate discrimination and may assist exploratory risk stratification and monitoring strategies. However, given the limitations of single-center data, limited sensitivity, lack of external validation, and potential calibration uncertainty, further prospective studies are required before clinical application.

## Data Availability

The raw data supporting the conclusions of this article will be made available by the authors, without undue reservation.
