# Supplementary material for: Development and validation of machine learning models for predicting pancreatic injury observed after tigecycline initiation: a retrospective real-world study
Source: Front Pharmacol. 2026 Sep 29;17:1930240. doi: 10.3389/fphar.2026.1930240 (PMC13658572; doi:10.3389/fphar.2026.1930240)
Supplement: Supplementary file 1 [file Supplementaryfile1.docx]

**Supplementary Material**

Supplementary Table S1: Clinical laboratory variables with the percentage of missing data. Pages 2

Supplementary Table S2: Comparison of model performance of different data imputation methods. Pages 3

Supplementary Table S3 Patient characteristics. Pages 4-5

Supplementary Table S4: Sensitivity analysis of KNN imputation methods with different hyperparameter K value. Pages 6

Supplementary Table S5: Sensitivity analysis of SMOTE oversampling with different K-neighbors value. Pages 7

Supplementary Table S6: Performance of LightGBM model with different feature combinations. Pages 8

Supplementary Table S7: The specific parameter Settings of the model implementation. Pages 9

Supplementary Figure S1: Sensitivity analysis in the data processing stage. Pages 10

Supplementary Figure S2: Top 21 features ranked by RF. Pages 11

Supplementary Figure S3: Five-fold cross-validation and ten-fold cross-validation of the LightGBM model. Pages 12

Supplementary Figure S4: SHAP dependence plot. Pages 13

**Supplementary Table S1: Clinical laboratory variables with the percentage of missing data**

| **Variables** | **Total (n)** | **Missing** | **Missing (%)** |
| --- | --- | --- | --- |
| ALB (g/L) | 623 | 0 | 0 |
| TBIL (μmol/L) | 623 | 0 | 0 |
| ALT (U/L) | 623 | 0 | 0 |
| AST (U/L) | 623 | 0 | 0 |
| eGFR (ml/min) | 623 | 89 | 14.29 |
| WBC (*10^9^/L) | 623 | 0 | 0 |
| NEU (*10^9^/L) | 623 | 0 | 0 |
| PLT (*10^9^/L) | 623 | 0 | 0 |
| LYM (*10^9^/L) | 623 | 0 | 0 |
| CRP (mg/L) | 623 | 0 | 0 |
| PCT (ng/ml) | 623 | 1 | 0.16 |
| GLU (mmol/L) | 623 | 104 | 16.69 |
| TC (mmol/L) | 623 | 144 | 23.11 |
| TG (mmol/L) | 623 | 144 | 23.11 |
| K (mmol/L) | 623 | 6 | 0.96 |
| Na (mmol/L) | 623 | 6 | 0.96 |
| PT (s) | 623 | 79 | 12.68 |
| APTT (s) | 623 |  |  |
| FIB (g/L) | 623 | 79 | 12.68 |
| D-dimer (μg/ml) | 623 | 82 | 13.16 |
| AMY (U/L) | 623 | 0 | 0 |
| LPS (U/L) | 623 | 0 | 0 |

ALB: albumin; TBIL: total bilirubin; ALT: alanine aminotransferase; AST: aspartate aminotransferase; eGFR: estimated glomerular filtration rate; WBC: white blood cell count; NEU: neutrophil count; PLT: platelet count; LYM: lymphocyte count; CRP: C-reactive protein; PCT: procalcitonin; GLU: glucose; TC: total cholesterol; TG: triglycerides; K: potassium; Na: sodium; PT: prothrombin time; APTT: activated partial thromboplastin time; FIB: fibrinogen; AMY: amylase; LPS: lipase.

**Supplementary Table S2: Comparison of model performance of different data imputation methods.**

| **Models** |  | **GBC** | **LightGBM** | **MLP** | **SVM** | **Voting classifier** | **XGBoost** | **RF** | **LR** |
| --- | --- | --- | --- | --- | --- | --- | --- | --- | --- |
| AUC | KNN imputation | 0.646 | 0.730 | 0.630 | 0.588 | 0.712 | 0.689 | 0.631 | 0.549 |
|  | Median imputation | 0.648 | 0.697 | 0.651 | 0.590 | 0.694 | 0.665 | 0.710 | 0.569 |
|  | Multiple imputation | 0.632 | 0.693 | 0.656 | 0.587 | 0.670 | 0.667 | 0.672 | 0.564 |
| Sensitivity | KNN imputation | 0.207 | 0.310 | 0.276 | 0.310 | 0.276 | 0.314 | 0.241 | 0.414 |
|  | Median imputation | 0.276 | 0.207 | 0.276 | 0.345 | 0.172 | 0.276 | 0.278 | 0.448 |
|  | Multiple imputation | 0.241 | 0.276 | 0.267 | 0.257 | 0.207 | 0.207 | 0.241 | 0.480 |
| Specificity | KNN imputation | 0.865 | 0.906 | 0.844 | 0.771 | 0.927 | 0.917 | 0.938 | 0.635 |
|  | Median imputation | 0.917 | 0.896 | 0.823 | 0.760 | 0.907 | 0.907 | 0.958 | 0.626 |
|  | Multiple imputation | 0.875 | 0.896 | 0.833 | 0.771 | 0.896 | 0.854 | 0.958 | 0.625 |
| PPV | KNN imputation | 0.316 | 0.500 | 0.348 | 0.290 | 0.533 | 0.529 | 0.538 | 0.255 |
|  | Median imputation | 0.500 | 0.375 | 0.320 | 0.303 | 0.367 | 0.471 | 0.667 | 0.265 |
|  | Multiple imputation | 0.368 | 0.444 | 0.333 | 0.266 | 0.375 | 0.300 | 0.636 | 0.280 |
| NPV | KNN imputation | 0.783 | 0.813 | 0.794 | 0.787 | 0.809 | 0.815 | 0.804 | 0.782 |
|  | Median imputation | 0.807 | 0.789 | 0.790 | 0.794 | 0.784 | 0.806 | 0.814 | 0.789 |
|  | Multiple imputation | 0.792 | 0.804 | 0.792 | 0.779 | 0.789 | 0.780 | 0.807 | 0.800 |
| Accuracy | KNN imputation | 0.712 | 0.768 | 0.712 | 0.664 | 0.776 | 0.779 | 0.756 | 0.584 |
|  | Median imputation | 0.768 | 0.736 | 0.696 | 0.664 | 0.736 | 0.760 | 0.800 | 0.584 |
|  | Multiple imputation | 0.728 | 0.752 | 0.704 | 0.656 | 0.736 | 0.704 | 0.792 | 0.592 |
| F1 | KNN imputation | 0.250 | 0.383 | 0.308 | 0.300 | 0.364 | 0.391 | 0.333 | 0.316 |
|  | Median imputation | 0.356 | 0.267 | 0.296 | 0.323 | 0.233 | 0.348 | 0.390 | 0.333 |
|  | Multiple imputation | 0.292 | 0.340 | 0.302 | 0.271 | 0.267 | 0.245 | 0.350 | 0.354 |

GBC: gradient boosting classifier; LightGBM: light gradient boosting machine; MLP: Multi-Layer Perceptron; SVM: support vector machine; XGBoost: eXtreme gradient boosting; RF: random forest; LR: logistic regression.

AUC: area under the receiver operating characteristic curve; NPV: negative predictive value; PPV: positive predictive value; F1: F1 score.

**Supplementary Table S3 Patient characteristics**

| Variables | Non-injury group (n=477) | Injury group (n=146) | P-value |
| --- | --- | --- | --- |
| Gender, n(%) |  |  | 0.975 |
| Male | 337(70.65%) | 104(71.23%) |  |
| Female | 140(29.35%) | 42(28.77%) |  |
| Age (years) | 56.0(46.0-69.0) | 59.0(47.25-69.0) | 0.542 |
| Smoke | 176(36.9%) | 57(39.04%) | 0.7109 |
| Drink | 145(30.4%) | 42(28.77%) | 0.7845 |
| Comorbidities |  |  |  |
| Hypertension | 223(46.75%) | 65(44.52%) | 0.705 |
| Diabetes | 114(23.9%) | 30(20.55%) | 0.466 |
| Hypertriglyceridemia | 46(9.64%) | 15(10.27%) | 0.948 |
| Fatty liver | 54(11.32%) | 19(13.01%) | 0.682 |
| Biliary tract stones | 66(13.84%) | 22(15.07%) | 0.812 |
| Cholecystitis | 42(8.81%) | 11(7.53%) | 0.755 |
| Concomitant medications |  |  |  |
| Diuretics | 370(77.57%) | 119(81.51%) | 0.369 |
| Glucocorticoids | 326(68.34%) | 118(80.82%) | 0.005 |
| Valproic Acid | 135(28.3%) | 41(28.08%) | 1 |
| DPP-4 inhibitors | 12(2.52%) | 1(0.68%) | 0.318 |
| Immunosuppressants | 14(2.94%) | 5(3.42%) | 0.7842 |
| Statins | 203(42.56%) | 66(45.21%) | 0.6386 |
| Biochemical indicators |  |  |  |
| Alb (g/L) | 34.8(31.0-37.8) | 34.2(30.55-37.78) | 0.33 |
| TBIL (μmol/L) | 11.0(6.9-20.7) | 12.0(8.02-23.4) | 0.2923 |
| ALT (U/L) | 25.0(12.0-51.0) | 22.0(12.0-48.5) | 0.7685 |
| AST (U/L) | 31.0(20.0-55.0) | 29.0(21.0-59.5) | 0.5767 |
| eGFR (ml/min) | 89.66(46.62-109.65) | 56.92(26.78-94.78) | ＜0.001 |
| WBC (*10^9^/L) | 9.92(6.53-14.56) | 11.16(7.36-16.32) | 0.0353 |
| NEU (*10^9^/L) | 7.98(5.05-12.14) | 9.25(6.1-13.95) | 0.0118 |
| PLT (*10^9^/L) | 181.0(86.0-284.0) | 160.0(92.25-249.0) | 0.1394 |
| LYM (*10^9^/L) | 0.88(0.53-1.37) | 0.86(0.48-1.31) | 0.65 |
| CRP (mg/L) | 76.88(34.51-135.36) | 85.2(43.95-159.0) | 0.0374 |
| PCT (ng/ml) | 0.59(0.18-1.78) | 1.32(0.42-5.9) | ＜0.001 |
| GLU (mmol/L) | 6.4(4.69-8.29) | 6.48(4.85-8.7) | 0.714 |
| TC (mmol/L) | 3.18(2.37-4.21) | 3.27(2.37-4.33) | 0.5379 |
| TG (mmol/L) | 1.63(1.1-2.6) | 1.69(1.27-2.29) | 0.6141 |
| K (mmol/L) | 4.08(3.71-4.45) | 4.11(3.73-4.5) | 0.4592 |
| Na (mmol/L) | 140.0(136.0-146.0) | 142.0(137.0-148.0) | 0.0289 |
| PT (s) | 13.2(12.1-14.35) | 13.0(12.0-14.0) | 0.4859 |
| APTT (s) | 28.9(25.8-33.2) | 30.15(26.6-33.98) | 0.0676 |
| FIB (g/L) | 4.41(3.22-5.94) | 4.13(3.06-5.66) | 0.4539 |
| D-dimer (μg/ml) | 4.53(2.54-8.57) | 4.25(2.38-8.35) | 0.7649 |
| AMY (U/L) | 64.0(41.0-99.0) | 67.0(48.25-98.75) | 0.2007 |
| LPS (U/L) | 47.0(25.0-99.0) | 60.5(31.5-106.0) | 0.0403 |
| Tigecycline |  |  |  |
| Loading dose (mg) | 100.0(100.0-100.0) | 100.0(100.0-100.0) | 0.2156 |
| Maintenance dose (mg) | 50.0(50.0-100.0) | 100.0(50.0-100.0) | 0.0032 |

ALB: albumin; TBIL: total bilirubin; ALT: alanine aminotransferase; AST: aspartate aminotransferase; eGFR: estimated glomerular filtration rate; WBC: white blood cell count; NEU: neutrophil count; PLT: platelet count; LYM: lymphocyte count; CRP: C-reactive protein; PCT: procalcitonin; GLU: glucose; TC: total cholesterol; TG: triglycerides; K: potassium; Na: sodium; PT: prothrombin time; APTT: activated partial thromboplastin time; FIB: fibrinogen; AMY: amylase; LPS: lipase.

**Supplementary Table S4: Sensitivity analysis of KNN imputation methods with different hyperparameter K value.**

| **Models** | **AUC** | **SE** | **SP** | **PPV** | **NPV** | **AC** | **F1** |
| --- | --- | --- | --- | --- | --- | --- | --- |
| K=1 | 0.690 | 0.207 | 0.906 | 0.400 | 0.791 | 0.744 | 0.273 |
| K=3 | 0.730 | 0.310 | 0.906 | 0.500 | 0.813 | 0.768 | 0.383 |
| K=5 | 0.685 | 0.241 | 0.854 | 0.333 | 0.788 | 0.712 | 0.280 |

AUC: area under the receiver operating characteristic curve; SE: sensitivity; SP: specificity; PPV: positive predictive value; NPV: negative predictive value; AC: accuracy; F1: F1 score

**Supplementary Table S5: Sensitivity analysis of SMOTE oversampling with different K-neighbors value.**

| **Models** | **AUC** | **SE** | **SP** | **PPV** | **NPV** | **AC** | **F1** |
| --- | --- | --- | --- | --- | --- | --- | --- |
| K=1 | 0.676 | 0.276 | 0.917 | 0.500 | 0.807 | 0.768 | 0.356 |
| K=3 | 0.730 | 0.310 | 0.906 | 0.500 | 0.813 | 0.768 | 0.383 |
| K=5 | 0.711 | 0.276 | 0.906 | 0.471 | 0.806 | 0.760 | 0.348 |

AUC: area under the receiver operating characteristic curve; SE: sensitivity; SP: specificity; PPV: positive predictive value; NPV: negative predictive value; AC: accuracy; F1: F1 score

**Supplementary Table S6: Performance of LightGBM model with different feature combinations.**

| **Models** | **AUC** | **SE** | **SP** | **AC** | **F1** |
| --- | --- | --- | --- | --- | --- |
| 40 | 0.730 | 0.310 | 0.906 | 0.768 | 0.383 |
| 37 | 0.709 | 0.276 | 0.917 | 0.771 | 0.356 |
| 34 | 0.696 | 0.241 | 0.906 | 0.752 | 0.311 |
| 31 | 0.711 | 0.207 | 0.896 | 0.736 | 0.267 |
| 28 | 0.676 | 0.241 | 0.875 | 0.728 | 0.292 |
| 25 | 0.715 | 0.310 | 0.927 | 0.784 | 0.400 |
| 22 | 0.718 | 0.276 | 0.896 | 0.752 | 0.340 |
| 19 | 0.723 | 0.310 | 0.906 | 0.768 | 0.383 |
| 16 | 0.682 | 0.207 | 0.885 | 0.728 | 0.261 |
| 13 | 0.697 | 0.345 | 0.865 | 0.744 | 0.384 |
| 10 | 0.721 | 0.414 | 0.875 | 0.768 | 0.453 |
| 7 | 0.726 | 0.379 | 0.833 | 0.728 | 0.393 |

LightGBM: light gradient boosting machine; AUC: area under the receiver operating characteristic curve; SE: sensitivity; SP: specificity; AC: accuracy; F1: F1 score.

**Supplementary Table S7: The specific parameter Settings of the model implementation.**

| Stages | Model parameter setting | Uses |
| --- | --- | --- |
| Data processing | KNNImputer(n_neighbors=3) | KNN imputation |
|  | SimpleImputer(strategy='median') | Median imputation |
|  | IterativeImputer(random_state=0) | Multiple imputation |
|  | StandardScaler() | Data standardization |
|  | SMOTE(k_neighbors=3) | Unbalanced data processing |
| Classification model | GradientBoostingClassifier (default parameters; random_state=42) | GBC |
|  | LGBMClassifier (default parameters; random_state=42) | LightGBM |
|  | MLPClassifier(max_iter=1000, random_state=42) | MLP |
|  | SVC(probability=True, random_state=42) | SVM |
|  | XGBClassifier(use_label_encoder=False, eval_metric='logloss', random_state=42) | XGBoost |
|  | LogisticRegression(max_iter=1000, random_state=42) | LR |
|  | RandomForestClassifier(default parameters; random_state=42) | RF |
|  | VotingClassifier(estimators=[('rf', RandomForestClassifier), ('xgb', XGBClassifier), ('lgbm', LGBMClassifier)], voting='soft' | Soft-voting ensemble of RF, XGBoost, and LightGBM |

**Supplementary Figure S1: Sensitivity analysis in the data processing stage.** (A) Performance metrics for the LightGBM model trained and run using datasets with different hyperparameter K value. (B) Performance metrics for the LightGBM model trained and run using datasets with different K-neighbors value. SMOTE: Synthetic Minority Over-sampling Technique; AUC: area under the receiver operating characteristic curve; NPV: negative predictive value.

**Supplementary Figure S2: Top 7 features ranked by RF.** PCT: procalcitonin; eGFR: estimated glomerular filtration rate; LPS: lipase; TC: total cholesterol; AMY: amylase; PLT: platelets.

**Supplementary Figure S3: Five-fold cross-validation and ten-fold cross-validation of the LightGBM model.** (A) Five-fold cross-validation of the final LightGBM model. (B) Ten-fold cross-validation of the final LightGBM model. ROC: receiver operating characteristic curve; AUC: area under the receiver operating characteristic curv

**Supplementary Figure S4: SHAP dependence plot**. Each dot represents a sample and each dependence plot shows how a single feature affects the output of the prediction model. SHAP: SHapley Additive exPlanation; PCT: procalcitonin; eGFR: estimated glomerular filtration rate; LPS: lipase; TC: total cholesterol; AMY: amylase; PLT: platelets.
